# Exploring Cognitive and Behavioral Changes Related to Smoking Among Medical Students in Saudi Arabia: A Cross-Sectional Study

**DOI:** 10.3390/medicina60121935

**Published:** 2024-11-24

**Authors:** Mohammed A. Aljunaid, Safa Abdullah Mehdar, Haneen Saud Bukhari, Rafal Hussain AlSharif, Rawaf Hussain AlSharif, Shahad AlHarbi

**Affiliations:** 1 Department of Family and Community Medicine, University of Jeddah, Jeddah 21589, Saudi Arabia; 2 Faculty of Medicine, University of Jeddah, Jeddah 21589, Saudi Arabia

**Keywords:** medical students, cognitive behavior, smoking, cessation, Saudi Arabia

## Abstract

*Background and Objectives:* This study aims to determine smoking patterns, prevalence, and associated factors among medical students in Jeddah, focusing on experimentation, daily smoking, cessation efforts, awareness of health risks, and attitudes toward quitting. *Materials and Methods:* A cross-sectional study was conducted in Jeddah, Saudi Arabia, from September to November 2023. It involved 213 medical students, from the second to sixth year, enrolled in various universities. Data collection utilized a comprehensive questionnaire adapted from the World Health Organization’s questionnaire and enhanced with additional relevant items by the researchers. The questionnaire, comprising eight sections, was administered online over a three-month period. *Results*: About 30.8% reported ever smoking, with 21.6% currently smoking. Among ever-smokers, 43.1% began smoking during college, mostly in the second year. Stress, exploration, and peer influence were frequent reasons for initiating smoking during college years. About 32.3% never considered quitting, while 67.7% thought about it. Regarding quit attempts, 53.8% attempted seriously, and 5.3% quit in the last year. Protective factors against smoking included health concerns and setting a good example. Most participants recognized smoking-related health hazards, with strong consensus on the effectiveness of anti-smoking measures. Notably, many participants expressed proactive attitudes toward smoking cessation interventions. *Conclusions*: Smoking is prevalent among Saudi medical students, often starting in college due to stress, new experiences, and peer influence. Many consider quitting, probably supported by the educational environment and a sense of responsibility toward personal and public health. Strengthened educational frameworks, including stress management training, are crucial for fostering a health-focused professional identity.

## 1. Introduction

Smoking poses a significant health risk, contributing to cardiovascular diseases, cerebrovascular accidents, and various cancers, notably lung cancer [1]. Given their pivotal role as future healthcare providers, medical students serve as influential figures, setting examples for patients and communities [2]. Research focused on medical students’ smoking habits is essential, particularly considering the stress they encounter during their academic progression stemming from peer comparison, demanding curricula, and licensure exams. Such stressors may lead to smoking as a coping mechanism, potentially exacerbating existing smoking habits [3,4,5,6]. Medical students are expected to adopt healthier lifestyles, both for their own well-being and to serve as role models for patients besides their crucial role in community education, smoking prevention, and cessation initiatives.

The prevalence of and factors related to smoking among medical students have been the subject of various studies, revealing a complex relationship between medical education and smoking behaviors and attitudes toward smoking. The Global Health Professions Student Survey indicated that smoking rates among medical students in Europe were generally higher than those in the general population, with rates around 30% [7]. Another study by Chkhaidze et al. reported smoking prevalence rates as high as 47% among medical students in Georgia, indicating that medical education does not universally deter smoking [8]. On the other hand, Afifah et al. noted that tobacco use was higher among final-year medical students compared to first-year students, suggesting that exposure to medical education does not necessarily prevent smoking but may instead reflect a cumulative effect over time [9]. These data suggest that medical students are at risk of adopting smoking behaviors during their college years, which is inconsistent with the expected impact of medical education. This is particularly concerning as smoking among healthcare professionals can negatively impact their ability to counsel patients effectively on smoking cessation [10]. These findings highlight the need to understand the mechanisms that induce smoking behaviors among medical students to inform targeted interventions beyond the scope of standard medical education.

However, there is limited research exploring the relationship between smoking behaviors and academic progression among medical students, notably in Saudi Arabia. Available studies focused on estimating the prevalence of smoking, showing several associations with psychosocial and academic determinants. A cross-sectional study conducted at King Fahad Medical City in Riyadh revealed that 39.8% of the participating students had smoked before, with 17.6% being current smokers. The mean age of smoking initiation was 15.8 (±3.3) years, with a significantly higher proportion of males being smokers than females. Primary reasons for smoking included leisure, imitation, and stress relief, while health and religious concerns motivated non-smokers [11]. A cross-sectional survey at Prince Sultan Military College of Health Sciences in Dammam demonstrated that smokers had lower grade point averages, increased absences, and more academic warnings, with a negative correlation between cigarette consumption and grade point averages [12]. Another study at the College of Medicine, Jazan University, found the highest smoking prevalence among third-year students (15.1%), contrasting with the lowest rates among second-year students (8.6%) [13].

Therefore, this study aims to determine the patterns, prevalence, and changes in smoking and smoking-related behaviors and their association with academic progression among medical students enrolled in prestigious institutions such as the University of Jeddah, King Abdulaziz University, Ibn Sina University, Batterjee Medical College, King Saud University, and Fakeeh College of Medical Sciences in Jeddah, Saudi Arabia. By elucidating this relationship, we seek to provide insights into potential interventions and educational programs tailored to mitigating smoking habits among medical students and future healthcare professionals. This study aimed to achieve the following objectives:

- To describe the changes in smoking behaviors among medical students during college years, including smoking experimentation and initiation, active smoking, and quit attempts.
- To explore the cognitive, psychosocial, and educational factors influencing smoking behaviors and quit attempts among medical students.
- To examine medical students’ attitudes toward their future role in proactive health interventions.

Such data provide insights into the cognitive and behavioral changes related to smoking among medical students and emphasize the role of medical education in smoking cessation and prevention.

## 2. Materials and Methods

### 2.1. Design

This cross-sectional study was conducted in Jeddah, Saudi Arabia, between September 2023 and November 2023. Ethical approval was obtained from the University of Jeddah (Ref# UJ-REC-146).

### 2.2. Participants

Inclusion criteria encompassed female and male medical students from the second to sixth years enrolled in specific universities: University of Jeddah, King Abdulaziz University, King Saud University, Ibn Sina National College for Medical Studies, Fakeeh College for Medical Science, and Batterjee Medical College. Exclusion criteria included interns and medical students from other universities. The first year was excluded because, in Saudi Arabia, the first year is considered a preparatory year and does not count as part of medical college. Students who successfully complete the preparatory year with a high GPA can apply to and join medical school. Therefore, the second year in medical school is considered the actual first year of medical education.

### 2.3. Data Collection

Data collection used an adapted version of the World Health Organization questionnaire on tobacco smoking for a survey among healthcare professionals [14]. Items were selected as adapted based on relevance with regards to the study objectives. Other questions were added by authors, notably, to explore how specific smoking behaviors evolved during the college years and other relevant dimensions. Thus, the administered questionnaire was organized into eight sections:

1. Demographic and academic data, including age, sex, nationality, academic year, etc.
2. Smoking experiences and behavior, including ever smoking, ever smoking daily for at least six months, current smoking frequency (no smoking, occasional, daily), and smoking methods.
3. Smoking during medical college years, including whether smoking started during college years, year of start, intention for a one-time experience, smoking amount compared to the past year, quit attempts and duration of quitting, and forecasted smoking habits in the future.
4. Reasons for smoking initiation during college years, such as academic stress, peer influence, etc. (six reasons). Answering options were “no association”, “associated”, “contributing cause”, or “major cause”.
5. Protective cognitive factors against smoking, such as the occurrence of health symptoms, protecting health, self-discipline, etc. Each item was introduced with the following question: “To what extent does the following constitute a valid reason for not smoking?” Answering options were: not a reason, low, moderate, and high extent.
6. Knowledge about smoking-related health hazards, including a list of 11 health conditions such as bladder cancer, coronary artery disease, lung cancer, etc. Each hazard was categorized by the respondents’ responses: “Don’t know”, “No association”, “Contributing cause”, “Associated with”, and “Major cause”.
7. Perceived effectiveness of different anti-smoking measures. This section assessed participants’ opinions on the effectiveness of four anti-smoking measures, including health warnings on cigarette packages, smoking restrictions in closed public places, increased tobacco product prices, and restrictions on smoking in hospitals. Responses ranged from “Strongly disagree” to “Strongly agree”.
8. Attitudes toward second-hand smoke and smoking cessation and opinions regarding the role of doctors in addressing smoking (five items). This section assessed the agreement level from “Strongly disagree” to “Strongly agree”.

### 2.4. Procedure

Data collection was conducted online via Google Forms. The survey link was distributed through students’ most popular social media platforms and WhatsApp groups with respect to the targeted universities and faculties. The link was active for a duration of three months. Participants were able to access and complete the survey using the provided link within this timeframe.

### 2.5. Statistical Methods

The survey data were extracted as an Excel file from the Google Forms platform. It was then transferred for statistical analysis. Statistical analysis used the Statistical Package of Social Sciences (SPSS), version 21. Descriptive statistics were used to compute frequencies and percentages of categorical variables and means and standard deviations of continuous variables.

## 3. Results

### 3.1. Demographic and Academic Data

A total of 213 individuals reached the link, but 2 individuals refused to participate, and 211 completed the questionnaire. The mean (SD) age of the participants was 22.57 (2.20) years, and 125 (59.2%) were female. The majority of students were in their fifth (28.9%) and sixth (38.4%) years. Jeddah University (29.4%), King Abdulaziz University (20.9%), and King Saud University in Jeddah (12.8%) were the most represented institutions (Table 1).

### 3.2. Ever Smoking and Current Smoking Status

Of the total participants, 65 (30.8%) reported having ever smoked; among these, 31 (47.7%) indicated they had smoked daily for at least six months. Currently, 25 (38.5%) of the 65 identify as daily smokers, 19 (29.2%) no longer smoke, and 21 (32.3%) consider themselves occasional smokers. Thus, the rate of current smoking is 21.6%. The most frequently reported smoking methods were e-cigarette and manufactured cigarettes, used by 13 (20.0%) and 19 (29.2%) students, respectively. However, 40 (61.5%) did not declare their smoking method (Table 2).

### 3.3. Changes in Smoking During College Years

Changes in smoking behaviors during the college years are presented in Table 2. Among the 65 individuals who reported ever smoking, 28 (43.1%) began during their college years, most commonly in the second year (13, 20.0%). This implies that 13.3% of students start smoking during their medical college years. In most of these instances (19/28, or 67.9%), the initiation of smoking was intended as a one-time experience.

Furthermore, 32.3% (*n* = 21) have never considered quitting smoking, while 67.7% (*n* = 44) have thought about it. Regarding serious quit attempts, 46.2% (*n* = 30) have not made one, whereas 53.8% (*n* = 35) have attempted to quit smoking seriously, resulting in varying abstinence duration.

Compared to last year, a small proportion (3.1%) have quit smoking, 6.2% smoke less, 18.5% maintain the same smoking level, 10.8% smoke more, and 61.5% did not respond to the question on changes in their smoking habits. Regarding future smoking habits, 55.4% (*n* = 36) of participants are certain they will not smoke daily, 26.2% (*n* = 17) believe they will probably smoke daily, and 18.5% (*n* = 12) are certain they will smoke daily.

### 3.4. Reasons for Smoking Initiation During College Years

Stress related to study/exams, exploring new experiences/trends, and family stress were identified as the primary reasons for initiating smoking during college years, reported by 50%, 35.7%, and 21.4% of participants, respectively. When these factors were not cited as primary causes, they were considered contributing factors, noted by 10.7%, 14.3%, and 28.6% of participants, respectively. On the other hand, peer pressure toward smoking and grieving a loved one were acknowledged as major causes by 17.9% and 14.3% and as contributing factors by 10.7% each, respectively (Table 3).

### 3.5. Protective Cognitive Factors Against Smoking

In assessing protective cognitive factors against smoking among 211 participants, this study found that the most compelling reasons for not smoking were related to health protection (90% high importance), setting a good example for children (69.2% high importance), and self-discipline (68.7% high importance). The occurrence of certain symptoms also played a significant role, with 57.8% of participants rating it as a highly protective factor against smoking. Factors such as saving money and the influence of professional colleagues not to smoke were deemed important by over half of the respondents. Detailed percentages across different levels of importance (low, moderate, high) and the categorization of other factors are presented in Table 4.

### 3.6. Knowledge About Health Hazards Related to Smoking

In exploring the perceptions regarding smoking-related health hazards, lung cancer was recognized as a major cause by the highest percentage of participants (91.5%), followed closely by chronic bronchitis (84.4%), laryngeal cancer (77.3%), coronary artery disease (72.5%), and oral cancer (72.0%). Peripheral vascular disease, leukoplakia, neonatal death, and any soft tissue lesion in the mouth or lip were less frequently identified as majorly caused by smoking, yet were recognized as associated or a major cause by over half of the respondents (Table 5). Overall, the majority (92.4%) strongly agreed that smoking is harmful to health, with 63.0% expressing very high concern and 24.2% fairly high concern about the detrimental effects of smoking on their health.

### 3.7. Perceived Effectiveness of Different Anti-Smoking Measures

Participants’ perceptions of the effectiveness of various anti-smoking measures reveal a strong consensus on their potential impact (Table 6). The majority of the participants strongly agreed that health warnings on cigarette packages (55.0%), restrictions on smoking in closed public places (63.0%), and sharp increases in tobacco product prices (68.7%) are effective strategies for smoking cessation. Additionally, the restriction of smoking in hospitals to designated smoking areas was also viewed favorably, with 60.2% expressing strong agreement with its effectiveness. Additionally, between 15.6% and 21.3% expressed moderate agreement with the effectiveness of these measures, whereas only a minority expressed disagreement.

### 3.8. Various Perceptions and Attitudes Toward Smoking Cessation and Exposure

One-third of the participants (35.1%) strongly believe that most smokers can quit if they truly wish to, and 40.3% somewhat agree with this statement. Notably, 70.6% find it bothersome to be around someone smoking. Despite medical advice, 54.5% strongly agree that most individuals will not cease smoking, reflecting skepticism about the efficacy of health professional interventions. Regarding their capability to counsel patients on quitting smoking, 42.2% feel their current knowledge is adequate. Moreover, 60.7% advocate for seizing every appropriate patient interaction as an opportunity to discourage smoking, underscoring a proactive stance on the part of healthcare professionals (Table 7).

## 4. Discussion

### 4.1. Summary of Findings

The current national study offers an in-depth exploration of smoking habits, attitudes, and perceptions among medical students, particularly focusing on the changes in smoking behavior during their medical college years. This investigation seeks to establish a comprehensive framework that integrates psychological, social, and educational factors influencing smoking habits. Key findings reveal that 30.8% of participants have experimented with smoking, with nearly half of these individuals having smoked daily for at least six months. Notably, 43.1% of ever-smokers commenced smoking during their college years, primarily driven by stress related to studies, the allure of new experiences, and family pressures. Concurrently, a significant proportion of smokers have contemplated quitting, with over half having attempted serious quit efforts. Current smoking status showed that 46 (21.6%) were active smokers, of whom 25 (11.8%) were daily smokers. On the other hand, health protection, exemplary behavior, and self-discipline serve as potent disincentives against smoking. Furthermore, the participants demonstrated a high level of awareness regarding smoking-related health risks, along with quite favorable perceptions of the effectiveness of anti-smoking measures. The diverse attitudes toward the feasibility of quitting and the importance of proactive health professional interventions highlight a nuanced understanding of the smoking dilemma within the medical student community. These findings not only reflect the cognitive and behavioral changes related to smoking but also emphasize the critical role of medical education in fostering a deeper understanding and proactive approach to smoking cessation and prevention among future physicians.

### 4.2. Smoking Experiences

Assessment of smoking experiences showed that 30.8% of the participating medical students had ever experienced smoking, while the rate of active smoking was 21.6%. This concords with the figures of smoking prevalence among Saudi adolescents and undergraduate students, ranging between 2.4% and 39.6%, as shown by a systematic review [15]. More specifically, studies involving medical students showed disparate figures, ranging from 2.9% in a national study from 2012 [16] to 29.8% in male medical students from Riyadh in 2016 [17]. However, daily smokers accounted for 54.3% of current smokers, while the remainder described themselves as occasional smokers. International figures show similar smoking rates (30–47%) among medical students that are relatively higher than those observed in the general population [7,8]. These high figures of smoking among medical students may indicate an adverse health lifestyle and should be prioritized in health promotion interventions. A study from Jeddah showed that smoking among medical students was associated with lower levels of health responsibility, physical activity, nutrition, and interpersonal relationships. The authors suggested that planning and prioritizing health-promoting activities for medical students is crucial, not only to improve their lifestyles and health but also to potentially support population health promotion programs, given their future roles as healthcare providers [2].

### 4.3. Smoking Initiation and Behaviors over the College Years

Smoking was initiated during college years in 43% of ever-smokers, with academic stress being the most common factor. Before discussing academic stressors, it is worth mentioning that early adulthood—corresponding to the age group of our study population—is increasingly associated with smoking initiation and the transition from occasional to regular smoking in the general population. A repeated cross-sectional study involving nearly 80,000 young adults aged 22 to 23 years observed a two-fold increase in the proportion of ever-smokers, from 21% in 2002 to 43% in 2017, alongside a rise in transition to daily smoking, from 39% to 56% [18]. However, due to the demanding nature of the medical curriculum, medical students are more exposed to continuous stress compared to other specialties [6], which may increase the risk of both smoking initiation and transition to daily smoking. Other sources of stress include psychosocial stress, such as family pressure, financial concerns, and emotional relationships, which all contribute to the stress load [5]. This stress load significantly impacts the student’s physical and psychological well-being in addition to their academic performance [3]. Among the deleterious consequences of continuous academic stress is smoking. A qualitative study from Riyadh, Saudi Arabia, explored the role of smoking in stress management among medical students, revealing a complex and paradoxical relationship between stress and smoking behaviors. The study showed that while medical students are aware of the ineffectiveness of smoking as a stress relief tool, many continue to use it as a temporary escape from stress. This behavior is supported by a combination of habituation, social dynamics, and the intense pressure of their studies rather than a belief in its efficacy as a stress management technique [4]. The high levels of stress endured during the medical college years highlight the need to modernize medical education by implementing curricular reforms to improve the mental health of medical students [19].

Among the non-stress-related reasons for smoking initiation during college years are the exploration of new experiences and trends and peer pressure toward smoking. These were reported as major or contributing factors by 50.0% and 28.6% of the concerned subgroup, respectively. The influence of peers is a well-established factor in smoking initiation, as documented extensively in the literature [20].

With regards to changes in smoking behavior, we observed an interesting trend where most smoking initiation cases occurred during the second year of college. Subsequently, the number of new smokers significantly dropped in the third year and the following years. Additionally, two-thirds of the smokers have contemplated quitting, 53.8% have made serious attempts to quit, and 55.4% predict they will not smoke daily in the future. The observed trends in smoking behavior during medical college years carry significant implications for intervention strategies. The peak in initiation during the second year suggests that this period may be particularly stressful or associated with other factors conducive to smoking initiation. Understanding the specific causes—whether academic pressures, social changes, or others—is crucial for targeted interventions. Nonetheless, these trends appear to contradict data from both local and international studies. A study from Jazan, Saudi Arabia, showed that second-year medical students had the lowest smoking rates compared to their peers [13]. Similarly, a study from China demonstrated that susceptibility to smoking followed a U-shaped curve with age, indicating an initial decrease followed by an increase as students aged [21].

The high rates of contemplation and quit attempts among students highlight a clear need for smoking cessation support. This need presents an opportunity for health promotion programs to offer structured cessation support during medical education. Peer education is an effective method tailored for medical students. Establishing support or mentorship groups within medical schools utilizing peers and faculty who have successfully quit can be highly effective. These programs not only provide educational and emotional support but also reinforce positive behavior changes and self-efficacy [15]. By fostering a supportive community and highlighting successful role models, medical schools can improve smoking cessation rates, promoting healthier lifestyles and better role models for future clinicians.

### 4.4. Protective Factors Against Smoking

Health protection, exemplary behavior for children and patients, and self-discipline were shown to be potent disincentives against smoking among the study participants. Furthermore, students showed high levels of awareness about the health hazards of smoking despite some diseases where awareness was relatively low. These levels of awareness are probably shaped by knowledge gained during the medical college years. Likewise, a study among medical students in Egypt showed high levels of awareness about smoking hazards and their future role in fighting against smoking in community training [22]. Arguably, the effectiveness of awareness about smoking health hazards in combating smoking is questionable. Krosnick et al. suggest that public awareness of the relative risk of smoking rather than the absolute risk would elicit more behavioral change and provide better protection against smoking. This concept relates to the extent to which smoking increases the probability of adverse health outcomes [23].

Evaluating the perceived effectiveness of anti-smoking measures, this study finds strong support for all explored measures as effective cessation strategies, notably restrictions in public places, price increases, and the restriction of smoking areas in hospitals. This is consistent with data from Egypt [22]. On the other hand, health warnings on cigarette packaging have shown mixed perceptions of effectiveness. Findings from an Australian study suggest that such warnings are relatively more effective in non-smokers, as they prevent smoking initiation. However, adolescents appear to be desensitized to these warnings [24].

Interestingly, the present study showed that smoking is not socially encouraged among peers; even friends who smoke are reluctant to offer cigarettes, acknowledging the harmful implications of smoking. This indicates a higher level of awareness about the negative aspects of smoking even among smokers themselves [4]. This indicates that awareness, attitudes, and cognitive protection against smoking are dynamic constructs shaped by the students’ medical knowledge, experiences, and progressive embodiment of their future roles as doctors.

Another protective dimension against smoking is the attitude toward doctors’ roles in addressing smoking. Although only 42.2% of the participants declared being sufficiently knowledgeable to conduct smoking cessation counseling, 60.7% stated they would engage in smoking-related discussions with their patients. Consistently, the previous study from Egypt showed that medical students have high levels of commitment to the role of physicians in smoking cessation, coupled with a recognition of the need for specialized training [22].

The endorsement of a future doctor’s role represents a critical dimension in the cognitive and behavioral determinants of smoking among medical students. However, role model endorsement, adequate formal training in smoking cessation, and promoting a healthy lifestyle and positive behavioral changes are all interconnected dimensions [2,22,25,26]. Therefore, enhancing medical curricula to include comprehensive smoking cessation training could significantly improve public health outcomes. Equipping future physicians with these skills not only prepares them to better address smoking behaviors but also strengthens their role as health advocates. Additionally, promoting physicians as role models for healthy behaviors can increase their impact on patient and community health, ultimately contributing to lower smoking rates and improved public health.

### 4.5. Implications for Action

Findings from the present study indicate that the unique pressures, stressors, and health education opportunities of medical education significantly shape students’ attitudes toward smoking, potentially influencing their initiation, continuation, or cessation of smoking in addition to their attitudes and perceptions toward smoking and smoking cessation. These findings provide valuable insights and guidance toward the development of targeted interventions for smoking prevention and cessation within the medical student population. Based on these insights, several implications for action emerge:

- Develop targeted educational programs and create support networks: Institutions should integrate tailored health and well-being education programs into the medical curriculum. Establishing support groups or mentorship programs within medical schools would help address smoking cessation, notably by leveraging peers and faculty members who have successfully quit smoking.
- Incorporate stress management training: Since medical training can be highly stressful, providing students with effective stress management techniques could reduce the likelihood of smoking as a stress relief measure.
- Promote smoke-free policies: Strengthen smoke-free campus policies in medical schools to reduce environmental triggers for smoking and normalize non-smoking behaviors.
- Facilitate access to cessation resources: Ensure that medical students have easy access to smoking cessation resources, such as nicotine replacement therapy, counseling services, and digital health tools, like apps, designed to support quitting efforts.
- Leverage role modeling: Encourage faculty and senior medical professionals to model non-smoking behavior and actively participate in anti-smoking advocacy, reinforcing the anti-smoking message within the medical community.
- Conduct regular surveys and assessments: Periodically assess medical students’ smoking habits and attitudes toward smoking. Use these data to identify trends, at-risk individuals, and the effectiveness of current educational strategies.

### 4.6. Limitations

The present study has some limitations that hinder the validity and generalizability of the findings. The cross-sectional design and self-assessment methods pose a high risk for information bias. Additionally, the small sample size and the limited representativeness of faculties outside Jeddah restrict the statistical power and generalizability of the findings to the national level.

## 5. Conclusions

Smoking is highly prevalent among Saudi medical students, with a notable initiation of smoking habits during the college years linked to academic stress, new experiences, and peer influences. Remarkably, a substantial number of students have considered quitting, highlighting a recognition of the health risks associated with smoking.

Despite the challenges, there is a robust inclination toward cessation, facilitated by the educational environment that enhances awareness of smoking’s health risks and promotes anti-smoking attitudes. This environment not only educates students about the dangers of smoking but also fosters a sense of responsibility toward personal and public health.

Overall, the findings advocate for strengthened educational frameworks that integrate comprehensive smoking cessation training, promote health-conscious role models, and support the development of medical students into health professionals who can advocate effectively against smoking and influence public health positively. Additionally, considering the integration of stress management training within these curricula is crucial, as it can enhance the mental well-being of students, enabling them to manage the high levels of stress often associated with medical education. This approach not only addresses immediate health behaviors but also fosters a more sustainable, health-focused professional identity.

## Figures and Tables

**Table 1:** Demographics and academic characteristics (*n* = 211).

Parameter	Level	Mean	SD
Age	(years)	22.57	2.20
Parameter	Level	Frequency	Percentage
Sex	Male	86	40.8
	Female	125	59.2
Nationality	Saudi	191	90.5
	Non-Saudi	20	9.5
Academic year	2nd	20	9.5
	3rd	22	10.4
	4th	27	12.8
	5th	61	28.9
	6th	81	38.4
Place of living	Villa	100	47.4
	Apartment	97	46.0
	Hut	5	2.4
	University Campus/dormitory	9	4.3
College	Jeddah University	62	29.4
	King Abdulaziz University	44	20.9
	King Saud University	27	12.8
	Other	78	36.9
Marital status	Single	201	95.3
	Married	9	4.3
	Divorced	1	0.5
Ever smoked	No	146	69.2
	Yes	65	30.8

**Table 2:** Smoking patterns and behaviors among ever-smokers (*n* = 65).

Characteristics		*n*	% from Ever-Smokers	% from Relevant Subgroup
Smoking experiences				
Ever smoked daily for at least 6 months	No	34	52.3	
	Yes	31	47.7	
Current smoking frequency	Not at all	19	29.2	
	Occasionally	21	32.3	
	Daily	25	38.5	
Smoking method ^a^	Not reported	40	61.5	
	Reported	25	38.5	
	Manufactured cigarettes	13	20.0	
	e-cigarette	19	29.2	
	Hand rolled tobacco	2	3.1	
	Hookah	2	3.1	
	Cigar	3	4.6	
	Pipefuls	1	1.5	
Smoking dynamics during college years				
Started during medical college years	No	37	56.9	
	Yes	28	43.1	
If yes, what year?	Preparatory/1st year	4	6.2	14.3 ^b^
	2nd year	13	20.0	46.4
	3rd year	5	7.7	17.9
	4th year	4	6.2	14.3
	5th year	2	3.1	7.1
Intended to be a one-time experience	No	9	13.8	32.1 ^b^
	Yes	19	29.2	67.9
Smoking compared to last year	Has quit	2	3.1	
	Smokes less	4	6.2	
	Smokes same amount	12	18.5	
	Smokes more	7	10.8	
	Not answered	40	61.5	
Ever thought of quitting smoking	No	21	32.3	
	Yes	44	67.7	
Ever made a serious quit attempt	No	30	46.2	
	Yes	35	53.8	
Duration of quitting smoking	Days	6	9.2	17.1 ^c^
	Weeks	8	12.3	22.9
	Months	18	27.7	51.4
	Years	3	4.6	8.6
Smoking habits in the future	Will most certainly not smoke daily	36	55.4	
	Will probably smoke daily	17	26.2	
	Will most certainly smoke daily	12	18.5	

^a^ A participant may report more than one smoking method. ^b^ Percentages calculated among participants who started smoking during college years (*n* = 28). ^c^ Percentages calculated among participants who declared having made serious quit attempts (*n* = 35).

**Table 3:** Reasons for smoking initiation during college years (*n* = 28).

Factor	No Association		Associated With		Contributing Cause		Major Cause		Not Answered	
	*n*	%	*n*	%	*n*	%	*n*	%	*n*	%
Stress related to study/exams	6	21.4	4	14.3	3	10.7	14	50.0	1	0.5
Family stress	10	35.7	4	14.3	8	28.6	6	21.4	0	0.0
Peer pressure	13	46.4	6	21.4	3	10.7	5	17.9	1	0.5
Exploring new experience/trend	6	21.4	6	21.4	4	14.3	10	35.7	2	7.1
Grieving a loved one	16	57.1	3	10.7	3	10.7	4	14.3	2	7.1
Other	11	5.2	3	1.4	1	0.5	4	1.9	9	4.3

**Table 4:** Protective cognitive factors against smoking (*n* = 211).

Factor	Not a Reason		Low		Moderate		High	
	*n*	%	*n*	%	*n*	%	*n*	%
Occurrence of certain symptoms	26	12.3	14	6.6	49	23.2	122	57.8
To protect your heath	3	1.4	6	2.8	12	5.7	190	90.0
To save money	24	11.4	33	15.6	44	20.9	110	52.1
Self-discipline	5	2.4	20	9.5	41	19.4	145	68.7
To comply with pressure from professional colleagues not to smoke	71	33.6	56	26.5	21	10	63	29.9
Not to create discomfort in nearby people	33	15.6	28	13.3	44	20.9	106	50.2
To set a good example for patients	21	10	34	16.1	31	14.7	125	59.2
To set a good example for health workers	28	13.3	36	17.1	26	12.3	121	57.3
To set a good example for children	17	8.1	23	10.9	25	11.8	146	69.2
To set a good example for adults in your social environment	26	12.3	32	15.2	30	14.2	123	58.3

Items presented in this table explored the level of perceived importance of the given reason/factor for not smoking; items were introduced by the following question: “To what extent does the following constitute a valid reason for not smoking?”

**Table 5:** Knowledge about smoking-related health hazards (*n* = 211).

Health Hazard	Don’t Know		No Association		Contributing Cause		Associated With		Major Cause	
	*n*	%	*n*	%	*n*	%	*n*	%	*n*	%
Bladder cancer	13	6.2	25	11.8	78	37.0	38	18.0	57	27.0
Coronary artery disease	2	0.9	3	1.4	45	21.3	8	3.8	153	72.5
Lung cancer	0	0	1	0.5	12	5.7	5	2.4	193	91.5
Chronic bronchitis	1	0.5	2	0.9	24	11.4	6	2.8	178	84.4
Oral cancer	5	2.4	1	0.5	43	20.4	10	4.7	152	72.0
Pulmonary emphysema	6	2.8	4	1.9	43	20.4	14	6.6	144	68.2
Laryngeal cancer	2	0.9	1	0.5	38	18.0	7	3.3	163	77.3
Peripheral vascular disease	7	3.3	7	3.3	65	30.8	24	11.4	108	51.2
Leukoplakia	21	10	12	5.7	62	29.4	32	15.2	84	39.8
Any soft tissue lesion (mouth/lip)	10	4.7	7	3.3	61	28.9	34	16.1	99	46.9
Neonatal death	20	9.5	15	7.1	42	19.9	34	16.1	100	47.4

**Table 6:** Perceived effectiveness of different anti-smoking measures (*n* = 211).

Measure	Strongly Disagree		Somewhat Disagree		Neither Agree nor Disagree		Somewhat Agree		Strongly Agree	
	*n*	%	*n*	%	*n*	%	*n*	%	*n*	%
The health warning on cigarette packages	12	5.7	18	8.5	20	9.5	45	21.3	116	55.0
Smoking in closed public places being restricted	10	4.7	7	3.3	21	10.0	40	19.0	133	63.0
The sharp increase in price of tobacco products	10	4.7	9	4.3	14	6.6	33	15.6	145	68.7
Smoking in hospitals being restricted to special smoking area	17	8.1	11	5.2	23	10.9	33	15.6	127	60.2

Participants rated their level of agreement with the effectiveness of each of the above smoking cessation measures.

**Table 7:** Attitudes and opinions regarding the role of doctors in addressing smoking (*n* = 211).

Measure	Strongly Disagree		Neither Agree nor Disagree		Somewhat Agree		Strongly Agree	
	*n*	%	*n*	%	*n*	%	*n*	%
Most smokers can stop if they want to	22	10.4	30	14.2	85	40.3	74	35.1
It is annoying to be around someone who is smoking	4	1.9	20	9.5	38	18	149	70.6
Most people will not give up smoking even if their doctor tells them	2	0.9	23	10.9	71	33.6	115	54.5
Your current knowledge is sufficient as a basis for counseling patients who want to stop smoking	8	3.8	41	19.4	73	34.6	89	42.2
At every contact with a patient where it would be natural to do so, you should dissuade them from smoking	8	3.8	24	11.4	51	24.2	128	60.7

## Data Availability

Data from this study are available upon reasonable request from the authors.

## References

[1] Reitsma M.B., Fullman N., Ng M., Salama J.S., Abajobir A., Abate K.S., Abbafati C., Abera S.F., Abraham B., Abyu G.Y. (2017). Smoking prevalence and attributable disease burden in 195 countries and territories, 1990–2015: A systematic analysis from the Global Burden of Disease Study 2015. Lancet.

[2] Alzahrani S.H., Malik A.A., Bashawri J., Shaheen S.A., Shaheen M.M., Alsaib A.A., Mubarak M.A., Adam Y.S., Abdulwassi H.K. (2019). Health-promoting lifestyle profile and associated factors among medical students in a Saudi university. SAGE Open Med..

[3] Ribeiro Í.J., Pereira R., Freire I.V., de Oliveira B.G., Casotti C.A., Boery E.N. (2018). Stress and quality of life among university students: A systematic literature review. Health Prof. Educ..

[4] Abouammoh N., Irfan F., AlFaris E. (2020). Stress coping strategies among medical students and trainees in Saudi Arabia: A qualitative study. BMC Med. Educ..

[5] Ragab E.A., Dafallah M.A., Salih M.H., Osman W.N., Osman M., Miskeen E., Taha M.H., Ramadan A., Ahmed M., Abdalla M.E. (2021). Stress and its correlates among medical students in six medical colleges: An attempt to understand the current situation. Middle East Curr. Psychiatry.

[6] Bamuhair S.S., Al Farhan A.I., Althubaiti A., Agha S., Rahman S.u., Ibrahim N.O. (2015). Sources of stress and coping strategies among undergraduate medical students enrolled in a problem-based learning curriculum. J. Biomed. Educ..

[7] Torre G.L., Kirch W., Bes–Rastrollo M., Ramos R., Czaplicki M., Gualano M.R., Boccia A., GHPSS Collaborative Group (2012). Tobacco use among medical students in Europe: Results of a multicentre study using the global health professions student survey. Public Health.

[8] Chkhaidze I., Maglakelidze N., Maglakelidze T., Khaltaev N. (2013). Prevalence of and factors influencing smoking among medical and non-medical students in Tbilisi, Georgia. J. Bras. Pneumol..

[9] Afifah N., Sari S.Y.I., Miftahurachman M. (2014). Health behavior among medical students at Universitas Padjadjaran. J. Pendidik. Kedokt. Indones. Indones. J. Med. Educ..

[10] Warren C.W., Sinha D.N., Lee J., Lea V., Jones N.R. (2011). Tobacco use, exposure to secondhand smoke, and cessation counseling among medical students: Cross-country data from the global health professions student survey (GHPSS), 2005–2008. BMC Public Health.

[11] Al-Kaabba A.F., Saeed A.A., Abdalla A.M., Hassan H.A., Mustafa A.A. (2011). Prevalence and associated factors of cigarette smoking among medical students at King Fahad Medical City in Riyadh of Saudi Arabia. J. Fam. Community Med..

[12] Alqahtani J.S., Aldhahir A.M., Alanazi Z., Alsulami E.Z., Alsulaimani M.A., Alqarni A.A., Alqahtani A.S., AlAyadi A.Y., Alnasser M., AlDraiwiesh I.A. (2023). Impact of smoking status and nicotine dependence on academic performance of health sciences students. Subst. Abus. Rehabil..

[13] Alkhalaf M., Suwyadi A., AlShamakhi E., Oribi H., Theyab Z., Sumayli I., Yassin A., Aqeeli A., Alqassim A. (2021). Determinants and prevalence of tobacco smoking among medical students at Jazan University, Saudi Arabia. J. Smok. Cessat.

[14] World Health Organization (1983). Guidelines for the Conduct of Tobacco-Smoking Surveys Among Health Professionals.

[15] Alasqah I., Mahmud I., East L., Usher K. (2019). A systematic review of the prevalence and risk factors of smoking among Saudi adolescents. Saudi Med. J..

[16] Al-Bedah A.M., Basahi J.A., Ahmed S.S., Mohamed N.A. (2012). Saudi Arabia global health professional students tobacco survey 2010–2011. Life Sci. J..

[17] Almutairi K.M. (2016). Predicting relationship of smoking behavior among male Saudi Arabian college students related to their religious practice. J. Relig. Health.

[18] Barrington-Trimis J.L., Braymiller J.L., Unger J.B., McConnell R., Stokes A., Leventhal A.M., Sargent J.D., Samet J.M., Goodwin R.D. (2020). Trends in the age of cigarette smoking initiation among young adults in the US from 2002 to 2018. JAMA Netw. Open.

[19] Slavin S.J., Schindler D.L., Chibnall J.T. (2014). Medical student mental health 3.0. Acad. Med..

[20] Leshargie C.T., Alebel A., Kibret G.D., Birhanu M.Y., Mulugeta H., Malloy P., Wagnew F., Ewunetie A.A., Ketema D.B., Aderaw A. (2019). The impact of peer pressure on cigarette smoking among high school and university students in Ethiopia: A systemic review and meta-analysis. PLoS ONE.

[21] Peng S., Yu L., Yang T., Wu D., Bottorff J.L., Barnett R., Jiang S. (2019). Susceptibility to smoking and determinants among medical students: A representative nationwide study in China. Tob. Induc. Dis..

[22] Shalaby S.F., Soliman M.A. (2019). Knowledge, attitude, and practice of medical students regarding smoking and substance abuse, Cairo University, Egypt. J. Egypt. Public Health Assoc..

[23] Krosnick J.A., Malhotra N., Mo C.H., Bruera E.F., Chang L., Pasek J., Thomas R.K. (2017). Perceptions of health risks of cigarette smoking: A new measure reveals widespread misunderstanding. PLoS ONE.

[24] Drovandi A., Teague P.A., Glass B., Malau-Aduli B. (2018). Australian school student perceptions of effective anti-tobacco health warnings. Front. Public Health.

[25] Bilgiç N., Günay T. (2018). Evaluation of effectiveness of peer education on smoking behavior among high school students. Saudi Med. J..

[26] Orsal O., Ergun A. (2021). The effect of peer education on decision-making, smoking-promoting factors, self-efficacy, addiction, and behavior change in the process of quitting smoking of young people. Risk Manag. Healthc. Policy.

